# Genome-Wide Identification, Evolution and Expression Analysis of the Grape (*Vitis vinifera* L.) Zinc Finger-Homeodomain Gene Family

**DOI:** 10.3390/ijms15045730

**Published:** 2014-04-03

**Authors:** Hao Wang, Xiangjing Yin, Xiaoqin Li, Li Wang, Yi Zheng, Xiaozhao Xu, Yucheng Zhang, Xiping Wang

**Affiliations:** 1 State Key Laboratory of Crop Stress Biology in Arid Areas, College of Horticulture, Northwest A&F University, Yangling 712100, Shaanxi, China; E-Mails: hao274143118@gmail.com (H.W.); yinxiangjingsmile@163.com (X.Y.); lixiaoqin2829@163.com (X.L.); valley2013060091@gmail.com (L.W.); xxz@nwsuaf.edu.cn (X.X.); 2 Key Laboratory of Horticultural Plant Biology and Germplasm Innovation in Northwest China, Ministry of Agriculture, Northwest A&F University, Yangling 712100, Shaanxi, China; 3 Boyce Thompson Institute for Plant Research, Cornell University, Ithaca, NY 14853, USA; E-Mail: b07010081@cau.edu.cn; 4 Department of Plant Pathology, University of Florida, Gainesville, FL 32611, USA; E-Mail: yuchengzhang@ufl.edu

**Keywords:** synteny analysis, phylogenetic analysis, gene expression, grape, zinc finger-homeodomain

## Abstract

Plant zinc finger-homeodomain (ZHD) genes encode a family of transcription factors that have been demonstrated to play an important role in the regulation of plant growth and development. In this study, we identified a total of 13 *ZHD* genes (*VvZHD*) in the grape genome that were further classified into at least seven groups. Genome synteny analysis revealed that a number of *VvZHD* genes were present in the corresponding syntenic blocks of *Arabidopsis*, indicating that they arose before the divergence of these two species. Gene expression analysis showed that the identified *VvZHD* genes displayed distinct spatiotemporal expression patterns, and were differentially regulated under various stress conditions and hormone treatments, suggesting that the grape *VvZHD*s might be also involved in plant response to a variety of biotic and abiotic insults. Our work provides insightful information and knowledge about the *ZHD* genes in grape, which provides a framework for further characterization of their roles in regulation of stress tolerance as well as other aspects of grape productivity.

## 1. Introduction

Transcription factors (TFs) have been shown to play important roles in the regulatory networks of numerous developmental processes in plants, for instance, MYB (v-myb avian myeloblastosis viral oncogene homolog) TFs can regulate fruit color [1,2]. Transcription factors can either activate or repress target genes through direct binding to gene regulatory elements or motifs. Accordingly, different TF families evolve unique DNA-binding domains that confer their binding specificity. One of the well characterized domains is the homeodomain (HD) that is composed of about 60 amino acids [3]. Several HD- containing proteins/TFs are known to play crucial roles in plant or animal development [4–7] but plants evolve specific HD-Zip TF family [8] that bear a unique leucine zipper domain at the *C*-terminal end. Zinc fingers serve as important motifs in many regulatory proteins [9,10], typically consisting of two pairs of conserved cysteine and/or histidine residues that associated with zinc ions [11]. Zinc fingers can be divided into different categories according to the nature, number, and spacing pattern of the zinc-binding residues. For instance, C_2_H_2_, C_2_C_2_ and C_3_H zinc fingers interact with one zinc ion, while the recently identified plant RING finger and the animal Lin-11/Isl-1/Mec-3 (LIM) domain interact with two zinc ions [12–16]. It has been shown that many Zn-finger TFs and the C_2_H_2_-type protein factors are involved in various stress signaling pathways [17–20].

A cluster of zinc finger-homeodomain (ZHD) proteins were first discovered in *Flaveria* as a regulator of the gene encoding C4 phosphoenolpyruvate carboxylase (PEPCase) [21]. Since then, ZHD proteins have been identified and characterized in many plants, including *Arabidopsis thaliana* [22], *Oryza sativa* [23], *Glycine max* [24] and *Triticum aestivum* [25]. In *Arabidopsis*, the first described example of a zinc finger (ZF) HD protein is involved in the regulation of floral development [26], and an *Arabidopsis* ZHD protein (*AtZHD1*) was subsequently reported to bind specifically to the promoter of *Early Response To Dehydration Stress 1* (*ERD1*). The expression of *AtZHD1* is inducible by dehydration, salt stress and abscisic acid (ABA) [27]. To date, 14 *Arabidopsis ZHD* genes have been identified [26,28]. The function of *ZHD* genes in other plants has also been elucidated recently. For instance, soybean *GmZF-HD1* and *GmZF-HD2* directly up-regulate a gene coding for calmodulin isoform 4 (*GmCaM4*) upon pathogen stimulation [24]. Four rice *ZHD* genes have also been implicated in gene regulation [23].

Although the function of several *ZHD* genes have been elucidated in *Arabidopsis* and other model species, their roles in fruit crops including grape remains largely unknown. Grape is one of the most economically important perennial fruit crops worldwide and has been extensively studied at the physiological and developmental levels. Grape was one of the first fleshy fruit species to have its genome fully sequenced [29]. With the release of the grape genome it is now possible to perform a genome-wide identification and characterization of the family. Here we report the identification of 13 *ZHD* genes using the grape genome sequence, their classification, and phylogenetic and syntenic analyses. We also present their expression profiles in six different tissues/organs, and describe the dynamics of the corresponding transcript levels in response to different phytohormone treatments and various abiotic and biotic stresses.

## 2. Results

### 2.1. Genome-Wide Identification of V. vinifera ZHD (VvZHD) Genes

**Figure 1.**
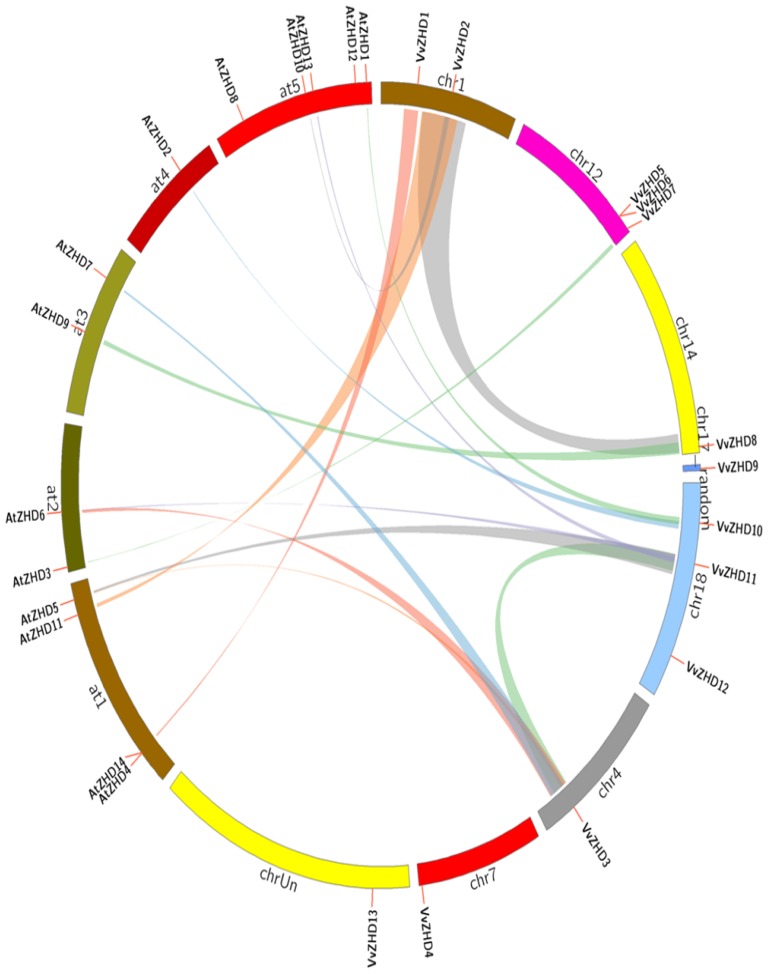
Synteny analyses of *VvZHD* genes and *ZHD* genes between grape and *Arabidopsis*. Grape and *Arabidopsis ZHD* genes are indicated by vertical orange lines. Colored bars denote syntenic regions.

**Figure 2.**
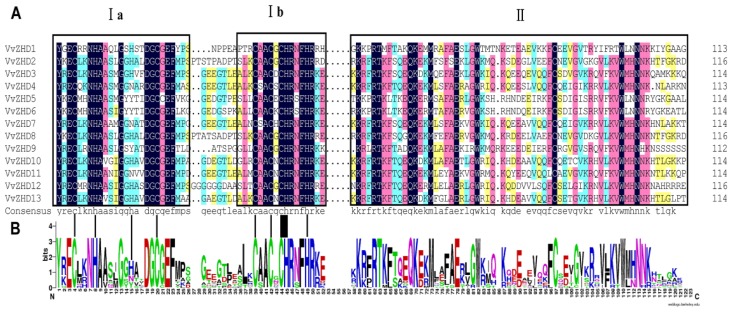
Alignment ZF and HD domains of VvZHD proteins. (**A**) A multiple sequence alignment of the ZF (Ia and Ib) and HD (II) domains of the grape ZHD proteins. The two conserved structures (Zinc-Finger and Homeodomain) are indicated; (**B**) Sequence logo of the ZF and HD domains of the grape ZHD proteins. The overall height of each stack represents the degree of conservation at this position, while the height of the individual letters within each stack indicates the relative frequency of the corresponding amino acids.

To identify ZHD genes in the grape genome, ZF and HD domains were used as a query to search the GenBank non-redundant protein database and the Grape Genome Database with the BLASTP program. After removing redundant sequences, we identified 13 putative *VvZHD* proteins with *E* values less than 0.01 (Table 1), all of which contain a complete HD and a ZF domain. All the genes were mapped to specific chromosomes and were named *VvZHD1*–*13* based on their order on the chromosomes (Figure 1). A multiple sequence alignment of the predicted full length protein sequences was carried out using the DNAMAN software to determine the domain structures of the VvZHD proteins. Two conserved domains were found (the zinc finger domain and homeodomain) in all the VvZHD proteins (Figure 2A) and they showed a high degree of sequence conservation within each class (Figure 2B).

### 2.2. Expansion Patterns of the Grape ZHD Gene Family

Segmental and tandem duplications are known to be the main causes leading to gene family expansions [30]. In the present study, we identified two tandemly duplicated ZHD genes (*VvZHD5* and *VvZHD6* located on grape chromosomes 12) (Figure 1). We also examined the segmentally duplicated blocks within the grape genome and identified two pairs of grape *ZHD* genes, *VvZHD2/8* and *VvZHD3/11*, which are associated with segmental duplications (Figure 1).

### 2.3. Evolutionary Relationship of Grape and Arabidopsis ZHD Genes

To explore the origins and evolutionary dynamics of the grape ZHD protein family, we performed a comparative synteny analysis between the grape and *Arabidopsis* genome sequences. Given that *Arabidopsis* ZHD genes have been well characterized [28], the knowledge generated in *Arabidopsis* could help determine the origin and diversification of *ZHD* genes in the grape. A large-scale syntenies containing 7 grape and 11 *Arabidopsis ZHD* genes were identified (Figure 1). Several grape orthologues including *VvZHD1*, *VvZHD7* and *VvZHD8* displayed synteny location with Arabidopsis *AtZHD14*, *AtZHD3*, *AtZHD9*, respectively. Interestingly, we also found that a single grape gene syntenically corresponded to multiple *Arabidopsis* genes such as *VvZHD2-AtZHD10/AtZHD11*, *VvZHD10-AtZHD1/AtZHD2*, *VvZHD11-AtZHD5/AtZHD6/AtZHD13* and *VvZHD3-AtZHD5/AtZHD6/AtZHD7*, suggestive of preferential gene deletion occurred in certain syntenic locations during grape genome evolution.

### 2.4. Phylogenetic Analysis of Grape ZHD Genes

**Figure 3.**
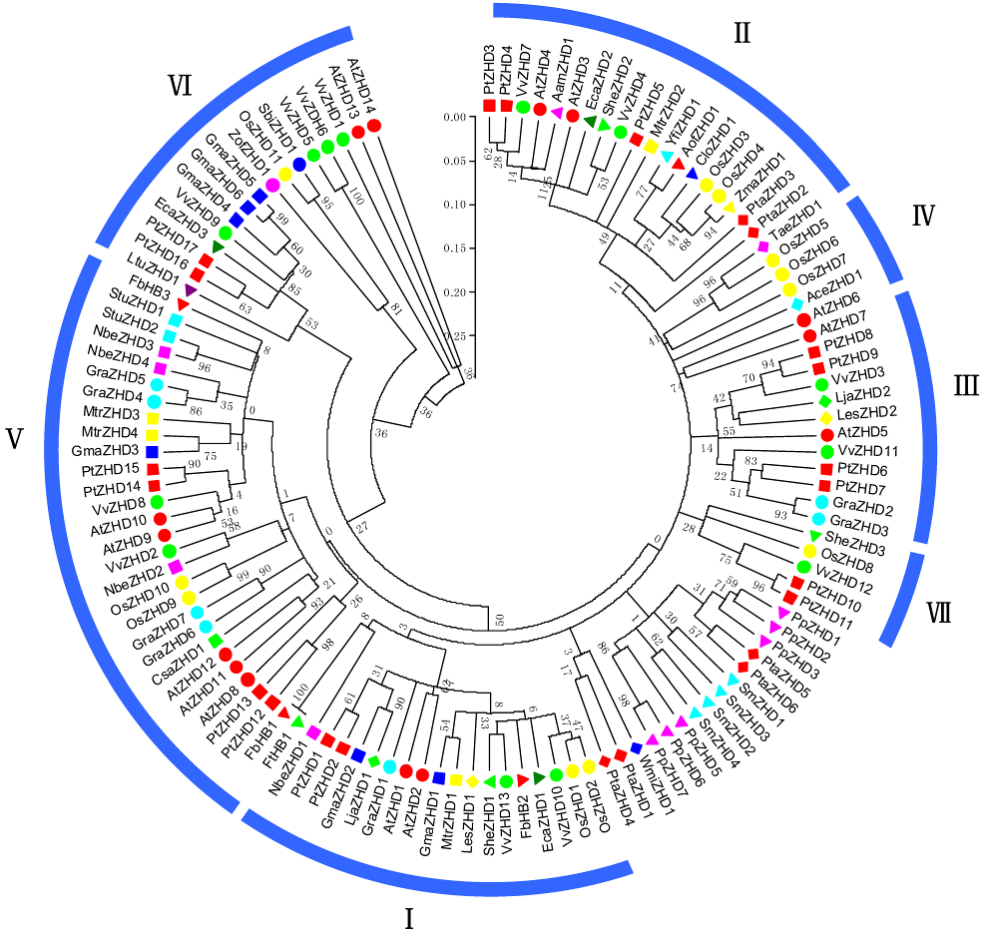
Phylogenetic analyses of plant ZHD proteins. Neighbor-joining (NJ) phylogenetic tree of 120 zinc finger-homeodomain (ZHD) proteins constructed with the zinc finger (ZF) and homeodomain (HD) domains.

Phylogenetic analyses of *ZHD* gene sequences from *Arabidopsis* and other plant species have previously been reported [28] and our result was in agreement with the earlier studies in general (Figure 3). The major clades of angiosperm *ZHD* genes have been shown to fall into class I to VII [28] while the *VvZHD* genes belong to classes I–III and V–VII (Figure 3). Class I contains two *VvZHD* genes (*VvZHD10 and 13*) and two *AtZHDs* share high levels of sequence similarity even outside the ZF and HD domains. Similarly, Class II includes two *VvZHD* genes (*VvZHD4 and 7*) as well as those from a wide range of angiosperms. Class III has two *VvZHD* genes (*VvZHD3 and 11*) that are fairly conserved at the *N*-terminal regions, whereas sequences in Class IV are not. Class IV *ZHD* genes encode proteins that are rich in glutamine (Q) at the *C*-termini, but there are no *VvZHD* genes in this clade. Class V includes two *VvZHD* genes (*VvZHD2* and *8*) and the central region of the encoded ZF domain in the corresponding proteins typically has more residues rather than are found in other ZHD proteins. Class VI, which has four *VvZHD* genes (*VvZHD1*, *5*, *6* and *9*) has the best supported major clade in the phylogenetic tree and the constituent sequences are highly divergent from the other classes (Figure 3). *VvZHD12* belongs to Class VII and shares no apparent sequence similarity with other ZHDs outside the ZF and HD domains.

### 2.5. Sequence Analysis of Grape ZHD Genes

**Figure 4.**
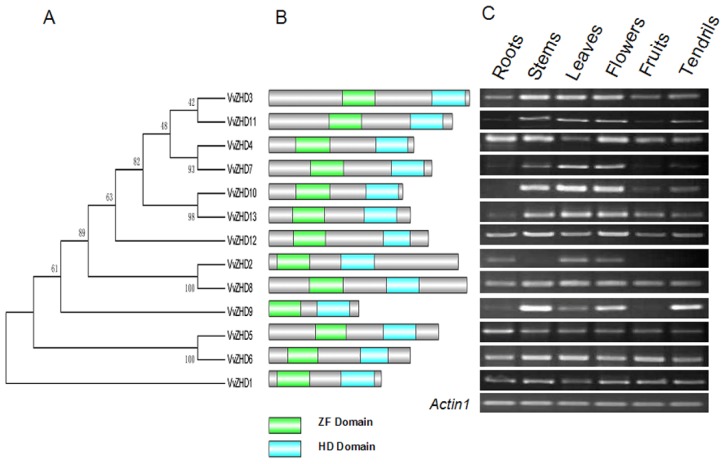
Phylogenetic relationships, primary protein structures and expression patterns of *VvZHD* genes. (**A**) Neighbor-joining un-rooted phylogenetic tree; (**B**) Schematic representation of protein length and domain location; (**C**) Expression analysis of *VvZHD* genes in various “Kyoho” (*V. labrusca* × *V. vinifera*) organs by semi-quantitative RT-PCR. *Actin1* was used as the internal control.

Phylogenetic relation among the 13 VvZHD proteins was analyzed and presented in Figure 4A. VvZHD1 appears to distantly relate to the rest of the genes. To delineate the evolutionary relationships among the *VvZHD* genes, we determined the distribution of the conserved domains in the encoded proteins (Figure 4B) and they all shared the following basic structure with ZF and HD domains (Figure 4B).

### 2.6. Expression Profiles of Grape ZHD Genes in Different Organs

To gain insights into the potential functions of the grape *ZHD* genes during development, we performed semi-quantitative RT-PCR analysis in various tissues. Figure 4C shows that all of the 13 genes were expressed in at least one of the six tissues but with various specificities. Most of the genes, including *VvZHD1*, *3*–*6*, *8* and *12*, were expressed in all tissues examined, while the remainders showed tissue-specific expression. However, ten *VvZHD* genes that were constitutively expressed in all organs exhibited notable variation in expression levels, with *VvZHD3* displaying high levels of expression in stems, leaves and flowers, but much lower expression in the roots, fruits and tendrils.

### 2.7. Expression Patterns of Grape ZHD Genes under Different Stress Conditions and Following Exogenous Hormone Treatments

Different approaches have been developed to improve plant stress tolerance, including manipulating and reprogramming the expression of endogenous stress-related genes. Therefore, identification and functional characterization of potential stress-related genes provides fundamental information for future improvement of plant stress tolerance. In the present study, we investigated the response of grape *ZHD* genes to various abiotic and biotic stress conditions, as well as hormone treatments.

#### 2.7.1. Abiotic Stresses

**Figure 5.**
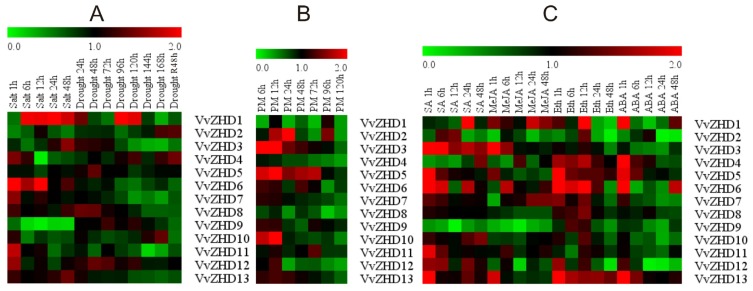
Expression profile of 13 *VvZHD* genes under salinity and drought stress treatments, powdery mildew inoculation and various hormone treatments (SA, MeJA, Eth, ABA) analyzed by semi-quantitative RT-PCR. Blocks with different colors indicate the transcript accumulation relative to the respective control: up-regulated (red), down-regulated (green). (**A**) Expression profile of *VvZHD* genes under biotic stress treatments, salinity and drought (original results shown in Figure S1); (**B**) Expression profile of *VvZHD* genes under biotic stress treatment, powdery mildew (original results shown in Figure S1); (**C**) Expression profile of *VvZHD* genes following four hormone treatments: SA, MeJA, Eth and ABA (original results shown in Figure S2).

**Figure 6.**
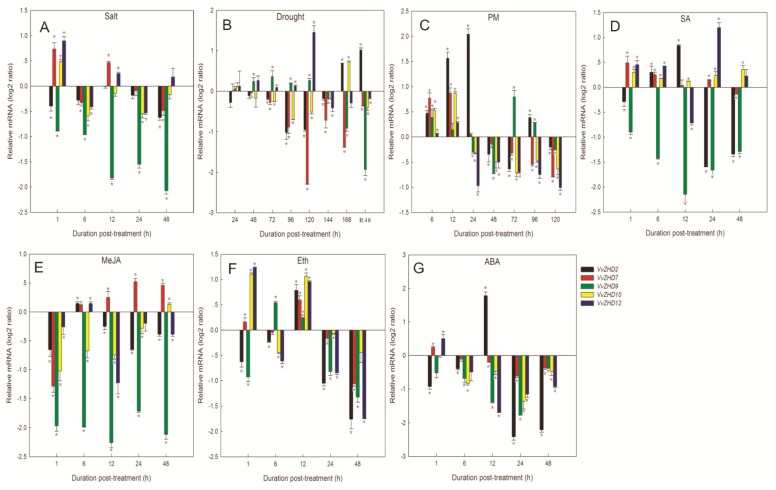
Effect of salt (**A**); drought (**B**); powdery mildew (PM) (**C**); salicylic acid (SA) (**D**); methyl jasmonate (MeJA) (**E**); ethylene (Eth) (**F**) and abscisic acid (ABA) (**G**) on the expression of *VvZHD* gene expression in leaves was investigated by qRT-PCR. The grape actin1 gene was used as the reference gene. Error bars represent SD (*n =* 3). The gene expression results were normalized to the mock controls, respectively. Asterisks indicate levels of significance of differential expression (*t*-test: * *p* ≤ 0.05).

To determine whether *VvZHD* genes respond to osmotic stress in leaves, semi-quantitative RT-PCR was performed to assess their transcript levels under salt and drought stress conditions. As shown in Figure 5, the expression of five *VvZHD* genes (*VvZHD1*, *3*, *6*, *8* and *12*) was differentially regulated by both treatments. In particular, the transcript level of *VvZHD11* was gradually down-regulated under drought stress, but increased after re-hydration, while *VvZHD5* did not respond to the treatment. *VvZHD6*, *7* and *13* were up-regulated in response to salt stress, but down-regulated by drought stress. The opposite response of salt stress was observed for *VvZHD2*, *3* and *9. VvZHD10* was transiently up-regulated in response to salt stress and then declined, but increased gradually with time under drought stress. *VvZHD5* did not respond to either treatment. The consistent results were obtained for five randomly selected *VvZHD* genes by real-time RT-PCR under the same treatments (Figure 6).

#### 2.7.2. Biotic Stress

The responses of the 13 *VvZHD* to powdery mildew infection were investigated by semi-quantitative RT-PCR. More than half of 13 *VvZHDs* responded to the infection, with *VvZHD2*, *3*, *5*–*7* and *13* being up-regulated and *VvZHD1*, *4*, *8* and *12* being down-regulated (Figure 5). The transcript abundance of *VvZHD6* in infected leaves peaked at 48 h, but that of *VvZHD5* increased sharply at 6 h and remained constant thereafter till it decreased at 96 and 120 h. These results were further validated by real-time quantitative RT-PCR (Figure 6).

#### 2.7.3. Hormone Treatments

We also evaluated the effects of ABA, salicylic acid (SA), methyl jasmonate (MeJA), and ethylene (Eth) on the *VvZHD* gene expression since SA, MeJA and Eth are involved in plant response to biotic stresses [31,32]. Analysis of transcript levels from “Kyoho” grape leaves sprayed with SA showed that seven *VvZHD* genes (*VvZHD3*, *5*, *6* and *10–13*) were up-regulated to different degrees upon treatment, whereas 2 *VvZHD* genes (*VvZHD4* and *9*) were down-regulated. The transcript level of *VvZHD2* was high at the first three sampling points following SA treatment, but declined thereafter, while *VvZHD7* and *8* expressions remained unchanged (Figure 5). Following treatment with MeJA, four *VvZHD* genes (*VvZHD1*, *3*, *6* and *13*) exhibited increased expression (Figure 5), while five genes (*VvZHD2*, *5*, *8*, *9* and *12*) were down-regulated. *VvZHD4* and *7* transcript abundance was low at the first two sampling points after MeJA treatment, but subsequently increased. No obvious changes were noted in the expression of the other two *VvZHD* genes (*VvZHD10 and 11*) analyzed (Figure 5). As was the case with the Eth treatment, most *VvZHD* genes also showed altered expression in leaves following treatment (Figure 5). Of these, *VvZHD4–6* and *13* were induced by, whereas *VvZHD2* was down-regulated. The *VvZHD7* transcript level was high at the first three sampling times, but decreased thereafter.

ABA is known to play a central role in plant responses to various abiotic stresses [33] and analysis of transcript levels from “Kyoho” leaves treated with exogenous ABA indicated that three *VvZHD* genes (*VvZHD4*, *5* and *13*) were up-regulated at different times following treatment (Figure 5). For example, the transcript levels of *VvZHD4* and *13* were induced at 1 h after treatment, while those of *VvZHD1*, *5* and *6* peaked at 1 h after treatment, and *VvZHD2* transcript abundance at 12 h post-treatment. Three of the analyzed genes (*VvZHD3*, *9* and *10*) exhibited decreased transcript levels, whereas expression of *VvZHD8* remained unchanged. These results were also supported by quantitative real-time RT-PCR analysis (Figure 6).

## 3. Discussion

*ZHD* genes, which play importantly regulatory roles in plants, are critical for both development and responses to environmental changes. However, little is known about the grape *ZHD* genes, and their evolution and function, even though grape is one of the most important fruit crops worldwide and various forms of stress have posed serious negative impacts on the production and quality of grape crops worldwide. Accordingly, in this study we aimed to identify and characterize the structure, evolution, expression and stress-related responses of the grape *ZHD* gene family.

### 3.1. Evolution of Grape VvZHD Genes

*ZHD* genes encode a family of specific TFs in all major groups of land plants, including various seed plants, the seedless vascular plant *Selaginella*, and the nonvascular plant *Physcomitrella*, but are absent in fungi, yeast, the chlorophyte green algae *Chlamydomonas* and *Volvox*, and prokaryotes, which suggests that although plant *ZHD* genes may be derived from a common ancestor, and appeared after the divergence of land plants, many have undergone lineage specific differentiation [28]. In this study, a total of 13 genes were identified in the grape genome and subjected to phylogenetic analysis. The plant *ZHD* genes were clustered into seven distinct groups (CI–CVII), as well as a cluster that does not form a well-supported group (Figure 3). The plant *ZHD* gene family has diversified through evolution and our work suggests that the family has expanded during angiosperm evolution. Angiosperm *ZHD* genes form six well supported clades (Classes I–VI) and one weakly supported clade (Class VII) (Figure 3) and the grape *ZHD* genes were clustered into six of the seven groups (CI, CII, CIII, CV, CVI and CVII). Furthermore, most *VvZHD* genes were more closely related to *Arabidopsis ZHD* genes, which is consistent with the fact that grape and *Arabidopsis* are eudicots and diverged more recently from a common ancestor.

### 3.2. Expansion and Synteny of the VvZHD Gene Family

It is known that tandem, segmental and whole genome duplications have played significant roles in the evolution of many organisms [34]. Based on comprehensive analysis of gene chromosomal locations, lengths, structures and sequence similarities we determined that one pair of *VvZHD* genes, (*VvZHD5* and *6*) likely arose by tandem duplication. Similarly, genome duplications also contributed to the expansion of the *ZHD* gene family in other plants, such as the three whole-genome duplication events in the *Arabidopsis* genome [35]. More than half of the *Arabidopsis ZHD* genes are thought to be generated by genome duplication [28,36]; however, since the grape genome has apparently not undergone any recent whole genome duplication events [29], segmental and tandem duplications would probably account for most of the expansions, although there is debate on the exact nature and timing of these events in grape [29,37]. In summary, tandem and segmental duplications have likely played critical roles in the expansion and evolution of the *VvZHD* gene family, resulting in their quantitative, structural and functional diversification.

Genomic comparison is a relatively rapid and effective way to transfer genomic knowledge acquired in one taxon, whose genome structure, function and/or evolution is better characterized, to a less-studied organism [38]. Thus, the putative functions of *VvZHD* genes can be inferred from comparisons with their respective orthologs in the model plant *Arabidopsis*. In this study, synteny analysis of the grape and *Arabidopsis* genomes indicated that three pairs of *ZHD* genes (*VvZHD1-AtZHD14*, *VvZHD7-AtZHD3* and *VvZHD8-AtZHD9*) are located in syntenic genomic regions (Figure 1). The situation with other genes was more complex, including four cases of one *VvZHD* gene corresponding to multiple *AtZHD* genes, and although six *VvZHD* genes could not be mapped to any syntenic blocks, we could not conclude that these genes did not share a common ancestor. Since the lineages that led to grape and *Arabidopsis* have undergone multiple rounds of significant chromosomal rearrangement and fusions, followed by selective gene loss, the identification of chromosomal syntenies can be somewhat obscured [39]. It may therefore be deduced that some of the grape and *Arabidopsis ZHD* genes arose from a common ancestor, while others did not. The first *Arabidopsis* ZHD gene to be characterized, *AtZHD1*, was found to be involved in the floral transition [26] and now the list of *Arabidopsis ZHD* genes that have been functionally characterized includes *AtZHD1* [27], *AtZHD5* [22] and *AtZHD12* [26]. Based on such studies we can infer possible functions. For example, *VvZHD11* is a homolog of *AtZHD5* that functions as regulating floral architecture and leaf development.

### 3.3. Spatial Expression Patterns of VvZHD Genes in Various Grape Tissues

There are a couple of resemblances in the spatial expression models of *VvZHD* genes and those from other plant species. For instance, the *AtZHD10* and *VvZHD8* proteins are closely related and this is also reflected by their presence within the same phylogenetic group (V) (Figure 3). *AtZHD10* is expressed preferentially in inflorescence and seed tissues [28], while the closely related *VvZHD8* is highly expressed in leaves, flowers and stems. Members of groups I and III also appear to have similar expression patterns to each other. Hu *et al*. reported that *AtZHD9* was expressed in all tested tissues/organs [28]. This is similar to the expression patterns observed for *VvZHD8*, an orthologous gene with *AtZHD9* (Figure 1). It therefore seems that members of individual ZHD groups and orthologous pairs have similar expression features, again indicating that there may be conserved functionality amongst members of the same groups and orthologous genes.

### 3.4. VvZHD Proteins Play Important Roles in a Range of Biological Processes

To gain further insights into the potential roles of the *VvZHD* genes, we analyzed their spatiotemporal expression patterns, as well as their responsiveness to various types of abiotic and biotic stresses and hormone treatments. Our results revealed that the *VvZHD* genes exhibit diverse expression patterns in six organs, indicating widespread roles in plant development. Genes that showed higher expression levels in one organ than in others may be more important. One such example is *VvZHD6*, which are expressed at high levels in fruits and thus may play a role in their development.

We also showed that five of the *VvZHD* genes exhibited substantial increases in their levels of expression in response to dehydration and/or high salinity (Figure 5), two of which (*VvZHD1* and *12*) were up-regulated by both treatments. This aligns with a previous report that the *Arabidopsis ZHD1* gene is induced by dehydration, salt stress and ABA [27]. Moreover, *AtZHD1* can interact with NAM/ATAF1, 2/CUC2 (NAC) proteins and the simultaneous overexpression of *AtZHD1* and *AtNAC* genes improved *Arabidopsis* drought stress tolerance [27]. Our results suggest that, like a number of *ZHD* genes previously described, the *VvZHD* genes may play important roles in protecting grape from damage caused by various types of abiotic stress.

Since plant hormones such as ABA, SA, MeJA and Eth have been reported to be involved in stress tolerance, we also analyzed the responsiveness of 13 *VvZHD* genes to phytohormone treatments (Figure 5). Our results showed varying patterns of differential expression in response to the plant hormones, which correlates well with previous findings from other plant species. For instance, Eth, SA, and MeJA coordinately play a critical role in biotic stress signaling upon pathogen infection [40], while ABA is extensively involved in responses to various biotic and abiotic stresses, including pathogen infection [41], water-deficit, cold, and osmotic stress [42]. *Arabidopsis*, *AtZHD1* is up-regulated by ABA [43–45] and similarly, we found that *VvZHD4* and *13* exhibited increased expression after treatment with exogenous ABA. Besides, the expression of *TaZHD1* also correlates with Eth, ABA and MeJA obviously [25]. Taken together, our analyses of the responses of *VvZHD* genes to different plant hormone treatments revealed potentially important targets for increasing the resistance of grape to stress conditions, and will provide the basis for guiding future studies of the functions of *VvZHD* genes and their associated signal transduction networks.

## 4. Experimental Section

### 4.1. Identification and Annotation of Grape ZHD (VvZHD) Genes

The HMM (Hidden Markov Model) profile of the ZF domain (Accession No. PF04770) was downloaded from the Pfam database [46]. The HD domain was determined based on previous reports, as it is not present in Pfam [21]. These domains were then used as a query using profile hidden Markov models to search the GenBank non-redundant protein database [47] and the Grape Genome Database [48] using the BLASTP program. All hits with an *E* value <0.01 were collected and the domains were manually checked in each identified *VvZHD* gene.

### 4.2. Determination of Chromosomal Location and Synteny Analysis

The *VvZHD* genes were positioned on the grape pseudo-molecules available at the Grape Genome Database (12 X). Tandemly duplicated *VvZHD* genes were defined as adjacent to homologous *ZHD* genes on the grape chromosomes or within a sequence distance of 50 kb [49], with no more than one intervening gene [50]. For synteny analysis, we used MCScan algorithm for detection of synteny and collinearity [51]. Synteny blocks within the grape genome and between the grape and *Arabidopsis* genomes were downloaded from the Plant Genome Duplication Database, and those containing *VvZHD* genes were identified (Original results shown in Tables S1 and S2).

### 4.3. Sequence Alignments, Phylogenetic Analysis and Domain Location of VvZHD Genes

The 13 *VvZHDs* were aligned using ClustalX, which can provide an integrated system for performing multiple sequence and profile alignments and analyzing the results [52] (The Percent identity of alignment shown in Table S3). To compare and define subgroups, we integrated conserved ZF and HD domains into this dataset. Phylogenetic trees were constructed with the MEGA 5.0 software using the neighbor-joining (NJ) method, and the bootstrap test was replicated 1000 times [53]. The 13 *VvZHD* genes with the complete ZF and HD domains were used in this study. Pfam domains and signal peptides were predicted using SMART (European Molecular Biology Laboratory, Heidelberg, Germany) [54,55]. The diagram of protein structures was constructed using DOG 1.0 software (Hefei National Laboratory for Physical Sciences at Microscale and School of Life Sciences, University of Science &Technology of China, Hefei, China) [56,57].

### 4.4. Plant Material

Grape organs (young roots, stems, leaves, tendrils, flowers at the fully opening stage, and fruits at 33 days post anthesis) were harvested from two year-old “Kyoho” (*V. labrusca* × *V. vinifera*) seedlings grown in the field. “Kyoho” was also used for high salt, drought stress, and exogenous hormone treatments. *V. quinquangularis* “Shang-24” was used for powdery mildew (*Erysiphe necator*) inoculation. Both grape species are maintained in the grape germplasm resource orchard of Northwest A&F University, Yangling, China (34°20′N, 108°24′E).

### 4.5. Abiotic, Hormone and Biotic Stress Treatment

For abiotic stress treatments, two year-old “Kyoho” grape seedlings planted in pots were irrigated with 2 dm^3^ 250 mM NaCl [58,59]. Following treatments for 1, 6, 12, 24 and 48 h, the fully unfolded young leaves were collected. Drought stress was induced by withholding water from “Kyoho” seedlings as previously described, with minor modification [60,61]. Briefly, young leaves of the seedlings were harvested at 24, 48, 72, 96, 120, 144 and 168 h post treatment. Subsequently, the stressed plants were re-watered to soil saturation and leaves were collected at 48 h after re-watering. For salt and drought stress, plants watered every three days were used as controls.

Hormone treatments were conducted by spraying young leaves with 100 μM SA [62,63] or 100 μM ABA [58,64] followed by sampling at 1, 6, 12, 24 and 48 h post-treatment. Leaves sprayed with sterile distilled water at the same time points were collected as the control.

Pathogen infection was carried out by inoculating the young leaves of “Shang-24” with powdery mildew as previously described with minor modifications [65]. Prior to inoculation, leaves were sprayed with sterile water, and leaves were harvested at 6, 12, 24, 48, 72, 96 and 120 h post-inoculation (Hpi). Control plants were sprayed with sterile water and not inoculated.

At each time point of each treatment, nine leaves from three separate plants were pooled and immediately frozen in liquid nitrogen and stored at −80 °C until use.

### 4.6. Semi-Quantitative RT-PCR and Real-Time PCR Analysis

Total RNA was extracted according to Zhang *et al*. [66], and treated with 10 units of RNase-free DNase I (TaKaRa Bio Inc., Dalian, China) to remove genomic DNA contamination. For each sample, 1 μg of total RNA was used to synthesize first-strand cDNA using SuperScriptII reverse transcriptase (Invitrogen). For the following experiments, the reverse transcription products were diluted six times. The concentration of the cDNA was adjusted according to a PCR product corresponding to the grape *Actin1* gene (GenBank Accession number AY680701), amplified with the primers F (5′-GAT TCT GGT GAT GGT GTG AGT-3′) and R (5′-GAC AAT TTC CCG TTC AGC AGT-3′). Gene-specific primers were designed for the 13 *VvZHD* genes including the complete ZF and HD domains (Table S4). For semi-quantitative reverse transcription-PCR (RT-PCR), a 20 μL reaction volume included 1 μL of cDNA template, 1.6 μL of gene-specific primers (1.0 μM), 9.8 μL PCR Master Mix (Tiangen Biotech Co. Ltd., Beijing, China) and 7.6 μL sterile distilled water. The PCR parameters were 95 °C for 3 min, followed by 25–35 cycles of 95 °C for 30 s, 58 °C for 30 s, 72 °C for 25 s, and a final step at 72 °C for 2 min. Each PCR reaction was replicated three times. The results of semi-quantitative RT-PCR were quantified using the Gene Tools software, and the log-transformed values of the relative transcript abundance of *VvZHD* genes under abiotic, hormone and biotic stress treatment compared to the control were used for hierarchical cluster analysis with Genesis software.

Quantitative real-time PCR analysis was conducted with an IQ5 real-time PCR instrument (Bio-Rad, Hercules, CA, USA). Each reaction was carried out in triplicate with a reaction volume of 20 μL containing 1.6 μL of gene-specific primers (1.0 μM), 1.0 μL of cDNA, 10 μL of SYBR green (TaKaRa Bio Inc., Dalian, China), and 7.4 μL sterile distilled water. The PCR parameters were 95 °C for 30 s, followed by 40 cycles of 95 °C for 5 s and 60 °C for 30 s. Relative expression levels were analyzed with the IQ5 software using the Normalized Expression method (the gene expression results were also normalized to the mock controls, respectively) and a student *t*-test performed using the SPSS software (SPSS 17.0^®^, Chicago, IL, USA).

## 5. Conclusions

*ZHD* genes encode a family of transcription factors that are exclusively present in plants. While significant progress has been made in identifying and characterizing *ZHD* genes in a number of plant species, little equivalent information is available for fruit crops. In this study, we identified a total of 13 *VvZHD* genes in the grape genome and developed a nomenclature based on their chromosome location, and demonstrated that segmental duplication has likely contributed to the expansion and evolution of the *VvZHD* gene family. Comparative synteny analysis between grape and *Arabidopsis* showed that several grape and *Arabidopsis ZHD* genes were located in syntenic regions, suggesting that they have common ancestors. Expression analysis of the *VvZHD* genes suggested that all 13 genes were functional and that a few may have specific roles in particular organs. Finally, many *VvZHD* genes were also found to be responsive to various abiotic and biotic stresses and hormone treatments. These studies provide a resource for future studies of *VvZHD* genes and may promote research aimed at revealing their role in grape stress tolerance. The results provide the basic information of the grape *VvZHD* genes, which should facilitate further research aiming at revealing the potential important functions of *VvZHD* genes in grape.

## Supplementary Information



## Figures and Tables

**Table 1.** The grape *ZHD* gene family. CDS, coding sequence.

Gene name	Gene locus	Accession number	Chromosome location	Strand	CDS (bp)	Protein (aa)
*VvZHD1*	-	XM_002281662.1	chr1:6167005...6167589	−	585	194
*VvZHD2*	GSVIVT01019981001	XM_002273766.1	chr1:12042374...12043354	−	981	326
*VvZHD3*	GSVIVT01018947001	XM_002266541.2	chr4:18452642...18453679	+	1038	345
*VvZHD4*	GSVIVT01000250001	XM_002267711.1	chr7:20539010...20539762	−	753	250
*VvZHD5*	GSVIVT01023289001	XM_002264255.1	chr12:20065353...20066231	+	879	292
*VvZHD6*	-	XM_002264214.1	chr12:20068964...20069695	−	732	243
*VvZHD7*	GSVIVT01023137001	XM_003633364.1	chr12:22175301...22176146	−	846	281
*VvZHD8*	GSVIVT01011413001	XM_002283497.2	chr14:29460743...29461768	+	1026	341
*VvZHD9*	GSVIVT01000386001	XM_002263430.1	chr17:random 525867...526334	+	468	155
*VvZHD10*	GSVIVT01009128001	XM_002285673.1	chr18:5343830...5344522	+	693	230
*VvZHD11*	GSVIVT01009721001	XM_002281335.2	chr18:10783147...10784097	+	951	316
*VvZHD12*	GSVIVT01012772001	XM_002276544.1	chr18:23508908...23509735	−	828	275
*VvZHD13*	GSVIVT01003614001	XM_003635009.1	chrUn:11277853...11280416	−	735	244

## Abbreviations

ABA: abscisic acid
SA: salicylic acid
Eth: ethylene
MeJA: methyl jasmonate
ORF: open reading frame
qRT-PCR: quantitative reverse transcription PCR
ZHD: zinc finger-homeodomain

## References

[1] Espley R.V., Hellens R.P., Putterill J., Stevenson D.E., Kutty-Amma S., Allan A.C. (2007). Red colouration in apple fruit is due to the activity of the MYB transcription factor, MdMYB10. Plant J.

[2] Czemmel S., Heppel S.C., Bogs J. (2012). R2R3 MYB transcription factors: Key regulators of the flavonoid biosynthetic pathway in grapevine. Protoplasma.

[3] Bürglin T.R.A. (1994). Comprehensive classification of homeobox genes. Guidebook to the Homeobox Genes.

[4] Williams R.W. (1998). Plant homeobox genes: many functions stem from a common motif. Bioessays.

[5] Ito M., Sato Y., Matsuoka M. (2002). Involvement of homeobox genes in early body plan of monocot. Int. Rev. Cytol.

[6] Akin Z.N., Nazarali A.J. (2005). Hox genes and their candidate downstream targets in the developing central nervous system. Cell. Mol. Neurobiol.

[7] Hunter C.S., Rhodes S.J. (2005). LIM-homeodomain genes in mammalian development and human disease. Mol. Biol. Rep.

[8] Ariel F.D., Manavella P.A., Dezar C.A., Chan R.L. (2007). The true story of the HD-Zip family. Trends Plant Sci.

[9] Takatsuji H. (1999). Zinc-finger proteins: The classical zinc finger emerges in contemporary plant science. Plant Mol. Biol.

[10] Krishna S.S., Majumdar I., Grishin N.V. (2003). Structural classification of zinc fingers: Survey and summary. Nucleic Acids Res.

[11] Klug A., Schwabe J.W. (1995). Protein motifs 5. Zinc fingers. FASEB J.: Off. Public. Feder. Am. Soc. Exp. Biol.

[12] Halbach T., Scheer N., Werr W. (2000). Transcriptional activation by the PHD finger is inhibited through an adjacent leucine zipper that binds 14-3-3 proteins. Nucleic Acids Res.

[13] Li J., Jia D., Chen X. (2001). HUA1, a regulator of stamen and carpel identities in Arabidopsis, codes for a nuclear RNA binding protein. Plant Cell.

[14] Kosarev P., Mayer K.F., Hardtke C.S. (2002). Evaluation and classification of RING-finger domains encoded by the Arabidopsis genome. Genome Biol.

[15] Englbrecht C.C., Schoof H., Bohm S. (2004). Conservation, diversification and expansion of C2H2 zinc finger proteins in the Arabidopsis thaliana genome. BMC Genomics.

[16] Yanagisawa S. (2004). Dof domain proteins: Plant-specific transcription factors associated with diverse phenomena unique to plants. Plant Cell Physiol.

[17] Sakamoto H., Maruyama K., Sakuma Y., Meshi T., Iwabuchi M., Shinozaki K., Yamaguchi-Shinozaki K. (2004). Arabidopsis Cys2/His2-type zinc-finger proteins function as transcription repressors under drought, cold, and high-salinity stress conditions. Plant Physiol.

[18] Mittler R., Kim Y., Song L., Coutu J., Coutu A., Ciftci-Yilmaz S., Lee H., Stevenson B., Zhu J.K. (2006). Gain- and loss-of-function mutations in Zat10 enhance the tolerance of plants to abiotic stress. FEBS Lett.

[19] Huang J., Yang X., Wang M.M., Tang H.J., Ding L.Y., Shen Y., Zhang H.S. (2007). A novel rice C2H2-type zinc finger protein lacking DLN-box/EAR-motif plays a role in salt tolerance. Biochim. Biophys. Acta.

[20] Xu D.Q., Huang J., Guo S.Q., Yang X., Bao Y.M., Tang H.J., Zhang H.S. (2008). Overexpression of a TFIIIA-type zinc finger protein gene ZFP252 enhances drought and salt tolerance in rice (Oryza sativa L.). FEBS Lett.

[21] Windhovel A., Hein I., Dabrowa R., Stockhaus J. (2001). Characterization of a novel class of plant homeodomain proteins that bind to the C4 phosphoenolpyruvate carboxylase gene of Flaveria trinervia. Plant Mol. Biol.

[22] Hong S.-Y., Kim O.-K., Kim S.-G., Yang M.-S., Park C.-M. (2011). Nuclear import and DNA binding of the ZHD5 transcription factor is modulated by a competitive peptide inhibitor in Arabidopsis. J. Biol. Chem.

[23] Figueiredo D.D., Barros P.M., Cordeiro A.M., Serra T.S., Lourenço T., Chander S., Oliveira M.M., Saibo N.J. (2012). Seven zinc-finger transcription factors are novel regulators of the stress responsive gene OsDREB1B. J. Exp. Bot.

[24] Park H.C., Kim M.L., Lee S.M., Bahk J.D., Yun D.J., Lim C.O., Hong J.C., Lee S.Y., Cho M.J., Chung W.S. (2007). Pathogen-induced binding of the soybean zinc finger homeodomain proteins GmZF-HD1 and GmZF-HD2 to two repeats of ATTA homeodomain binding site in the calmodulin isoform 4 (GmCaM4) promoter. Nucleic Acids Res.

[25] Abu-Romman S. (2014). Molecular cloning and expression analysis of zinc finger-homeodomain transcription factor *TaZFHD1* in wheat. S. Afr. J. Bot.

[26] Tan Q.K.-G., Irish V.F. (2006). The Arabidopsis zinc finger-homeodomain genes encode proteins with unique biochemical properties that are coordinately expressed during floral development. Plant Physiol.

[27] Tran L.S.P., Nakashima K., Sakuma Y., Osakabe Y., Qin F., Simpson S.D., Maruyama K., Fujita Y., Shinozaki K., Yamaguchi-Shinozaki K. (2007). Co-expression of the stress-inducible zinc finger homeodomain ZFHD1 and NAC transcription factors enhances expression of the ERD1 gene in Arabidopsis. Plant J.

[28] Hu W., dePamphilis C.W., Ma H. (2008). Phylogenetic analysis of the plant-specific zinc finger-homeobox and mini zinc finger gene families. J. Integr. Plant Biol.

[29] Jaillon O., Aury J.M., Noel B., Policriti A., Clepet C., Casagrande A., Choisne N., Aubourg S., Vitulo N., Jubin C. (2007). The grapevine genome sequence suggests ancestral hexaploidization in major angiosperm phyla. Nature.

[30] Cannon S.B., Mitra A., Baumgarten A., Yong N.D., May G. (2004). The roles of segmental and tandem gene duplication in the evolution of large gene families in*Arabidopsis thaliana*. BMC Plant Biol.

[31] Fujita M., Fujita Y., Noutoshi Y., Takahashi F., Narusaka Y., Yamaguchi-Shinozaki K., Shinozaki K. (2006). Crosstalk between abiotic and biotic stress responses: A current view from the points of convergence in the stress signaling networks. Curr. Opin. Plant Biol.

[32] Huang D.Q., Wu W.R., Abrams S.R., Cutler A.J. (2008). The relationship of drought-related gene expression in Arabidopsis thaliana to hormonal and environmental factors. J. Exp. Bot.

[33] Finkelstein R.R., Gampala S.S.L., Rock C.D. (2002). Abscisic acid signaling in seeds and seedlings. Plant Cell.

[34] Xu G., Guo C., Shan H., Kong H. (2012). Divergence of duplicate genes in exon-intron structure. Proc. Natl. Acad. Sci. USA.

[35] Maere S., de Bodt S., Raes J., Casneuf T., van Montagu M., Kuiper M., van de Peer Y. (2005). Modeling gene and genome duplications in eukaryotes. Proc. Natl. Acad. Sci. USA.

[36] Blanc G., Hokamp K., Wolfe K.H. (2003). A recent polyploidy superimposed on older large-scale duplications in the Arabidopsis genome. Genome Res.

[37] Velasco R., Zharkikh A., Troggio M., Cartwright D.A., Cestaro A., Pruss D., Pindo M., FitzGerald L.M., Vezzulli S., Reid J. (2007). A high quality draft consensus sequence of the genome of a heterozygous grapevine variety. PLoS One.

[38] Lyons E., Pedersen B., Kane J., Alam M., Ming R., Tang H., Wang X., Bowers J., Paterson A., Lisch D. (2008). Finding and comparing syntenic regions among Arabidopsis and the outgroups papaya, poplar, and grape: CoGe with rosids. Plant Physiol.

[39] Zhang Y.C., Gao M., Singer S.D., Fei Z.J., Wang H., Wang X.P. (2012). Genome-wide identification and analysis of the TIFY gene family in grape. PLoS One.

[40] Glazebrook J. (2005). Contrasting mechanisms of defense against biotrophic and necrotrophic pathogens. Annu. Rev. Phytopathol.

[41] Mauch-Mani B., Mauch F. (2005). The role of abscisic acid in plant-pathogen interactions. Curr. Opin. Plant Biol.

[42] Davies W.J., Jones H.G. (1991). Abscisic Acid: Physiology and Biochemistry.

[43] Huang W.Z., Ma X.R., Wang Q.L., Gao Y.F., Xue Y., Niu X.L., Yu G.R., Liu Y.S. (2008). Significant improvement of stress tolerance in tobacco plants by overexpressing a stress-responsive aldehyde dehydrogenase gene from maize (Zea mays). Plant Mol. Biol.

[44] Kotchoni S.O., Kuhns C., Ditzer A., Kirch H.H., Bartels D. (2006). Over-expression of different aldehyde dehydrogenase genes in Arabidopsis thaliana confers tolerance to abiotic stress and protects plants against lipid peroxidation and oxidative stress. Plant Cell Environ.

[45] Rodrigues S.M., Andrade M.O., Gomes A.P.S., DaMatta F.M., Baracat-Pereira M.C., Fontes E.P.B. (2006). Arabidopsis and tobacco plants ectopically expressing the soybean antiquitin-like ALDH7 gene display enhanced tolerance to drought, salinity, and oxidative stress. J. Exp. Bot.

[46] Wellcome Trust Sanger Institute http://www.sanger.ac.uk.

[47] HMMER http://hmmer.janelia.org/search/hmmsearch.

[48] Genome Database for Rosaceae http://www.rosaceae.org/projects/grape_genome.

[49] Riechmann J.L., Heard J., Martin G., Reuber L., Jiang C.Z., Keddie J., Adam L., Pineda O., Ratcliffe O.J., Samaha R.R. (2000). Arabidopsis transcription factors: Genome-wide comparative analysis among eukaryotes. Science.

[50] Zhang Y.C., Mao L.Y., Wang H., Brocker C., Yin X.J., Vasiliou V., Fei Z.J., Wang X.P. (2012). Genome-wide identification and analysis of grape aldehyde dehydrogenase (ALDH) gene superfamily. PLoS One.

[51] Wang Y., Tang H., DeBarry J.D., Tan X., Li J., Wang X., Lee T.-h., Jin H., Marler B., Guo H. (2012). MCScanX: A toolkit for detection and evolutionary analysis of gene synteny and collinearity. Nucleic Acids Res.

[52] Thompson J.D., Gibson T.J., Plewniak F., Jeanmougin F., Higgins D.G. (1997). The CLUSTAL_X windows interface: Flexible strategies for multiple sequence alignment aided by quality analysis tools. Nucleic Acids Res.

[53] Tamura K., Peterson D., Peterson N., Stecher G., Nei M., Kumar S. (2011). MEGA5: Molecular evolutionary genetics analysis using maximum likelihood, evolutionary distance, and maximum parsimony methods. Mol. Biol. Evol.

[54] Letunic I., Doerks T., Bork P. (2012). SMART 7: Recent updates to the protein domain annotation resource. Nucleic Acids Res.

[55] SMART http://smart.embl-heidelberg.de/smart/set_mode.cgi?NORMAL=1.

[56] Ren J., Wen L.P., Gao X.J., Jin C.J., Xue Y., Yao X.B. (2009). DOG 1.0: Illustrator of protein domain structures. Cell Res.

[57] DOG 1.0—Protein Domain Structure Visualization http://dog.biocuckoo.org/.

[58] Boneh U., Biton I., Zheng C.L., Schwartz A., Ben-Ari G. (2012). Characterization of potential ABA receptors in Vitis vinifera. Plant Cell Rep.

[59] Upreti K.K., Murti G.S.R. (2010). Response of grape rootstocks to salinity: Changes in root growth, polyamines and abscisic acid. Biol. Plant.

[60] Cramer G.R., Ergul A., Grimplet J., Tillett R.L., Tattersall E.A.R., Bohlman M.C., Vincent D., Sonderegger J., Evans J., Osborne C. (2007). Water and salinity stress in grapevines: Early and late changes in transcript and metabolite profiles. Funct. Integr. Genomics.

[61] Yang Y.Z., He M.Y., Zhu Z.G., Li S.X., Xu Y., Zhang C.H., Singer S.D., Wang Y.J. (2012). Identification of the dehydrin gene family from grapevine species and analysis of their responsiveness to various forms of abiotic and biotic stress. BMC Plant Biol.

[62] Li H.E., Xu Y., Xiao Y., Zhu Z.G., Xie X.Q., Zhao H.Q., Wang Y.J. (2010). Expression and functional analysis of two genes encoding transcription factors, VpWRKY1 and VpWRKY2, isolated from Chinese wild Vitis pseudoreticulata. Planta.

[63] Wang L.J., Li S.H. (2006). Thermotolerance and related antioxidant enzyme activities induced by heat acclimation and salicylic acid in grape (*Vitis vinifera* L.) leaves. Plant Growth Regul.

[64] Xiao H.G., Nassuth A. (2006). Stress- and development-induced expression of spliced and unspliced transcripts from two highly similar dehydrin 1 genes in V-riparia and V-vinifera. Plant Cell Rep.

[65] Wang Y., Liu Y., He P., Chen J., Lamikanra O., Lu J. (1995). Evaluation of foliar resistance to uncinula necator in chinese wild vitis species. Vitis.

[66] Zhang J.J., Wang Y.J., Wang X.P., Yang K.Q., Yang J.X. (2003). An improved method for rapidly extracting total RNA from Vitis. Fruit Sci.

