# Supplementary material for: Genome-Wide Identification, Evolution and Expression Analysis of the Grape (Vitis vinifera L.) Zinc Finger-Homeodomain Gene Family
Source: Int J Mol Sci. 2014 Apr 3;15(4):5730–48. doi: 10.3390/ijms15045730 (PMC4013592; doi:10.3390/ijms15045730)
Supplement: Supplementary file 1 [file ijms-15-05730-s001.pdf]

Supplementary Information

Figure S1. Expression profiles of 13 *VvZHD* genes under drought stress, salinity stress treatment and powdery mildew (*Erysiphe necator*) inoculation analyzed using semi-quantitative RT-PCR. *Actin1* (GenBank Accession number AY680701) was used as an internal control. The upper and lower bands represent treatment and control, respectively.

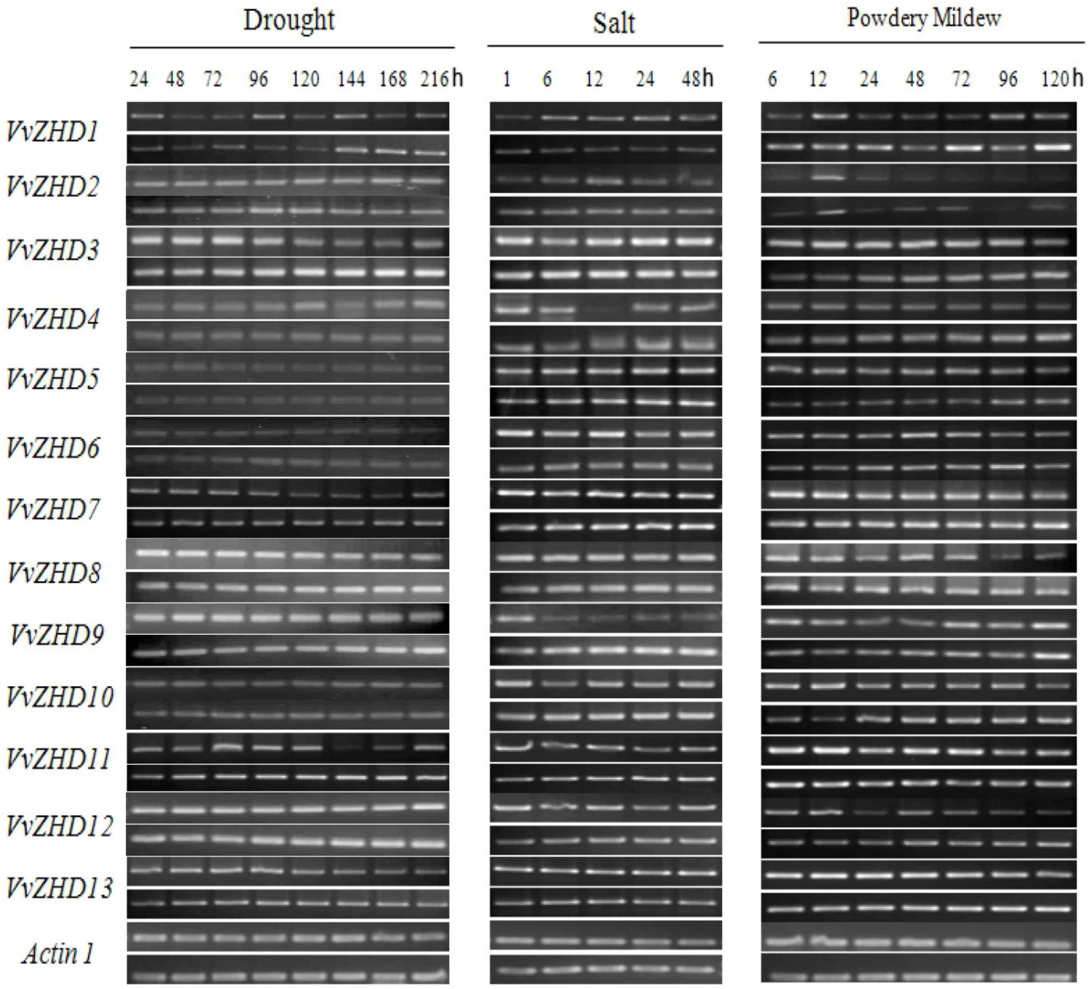

Figure S2. Expression profiles of 13 *VvZHD* genes under SA, MeJA, Eth and ABA treatment analyzed using semi-quantitative RT-PCR. *Actin1* (GenBank Accession number AY680701) was used as an internal control. The upper and lower bands represent treatment and control, respectively.

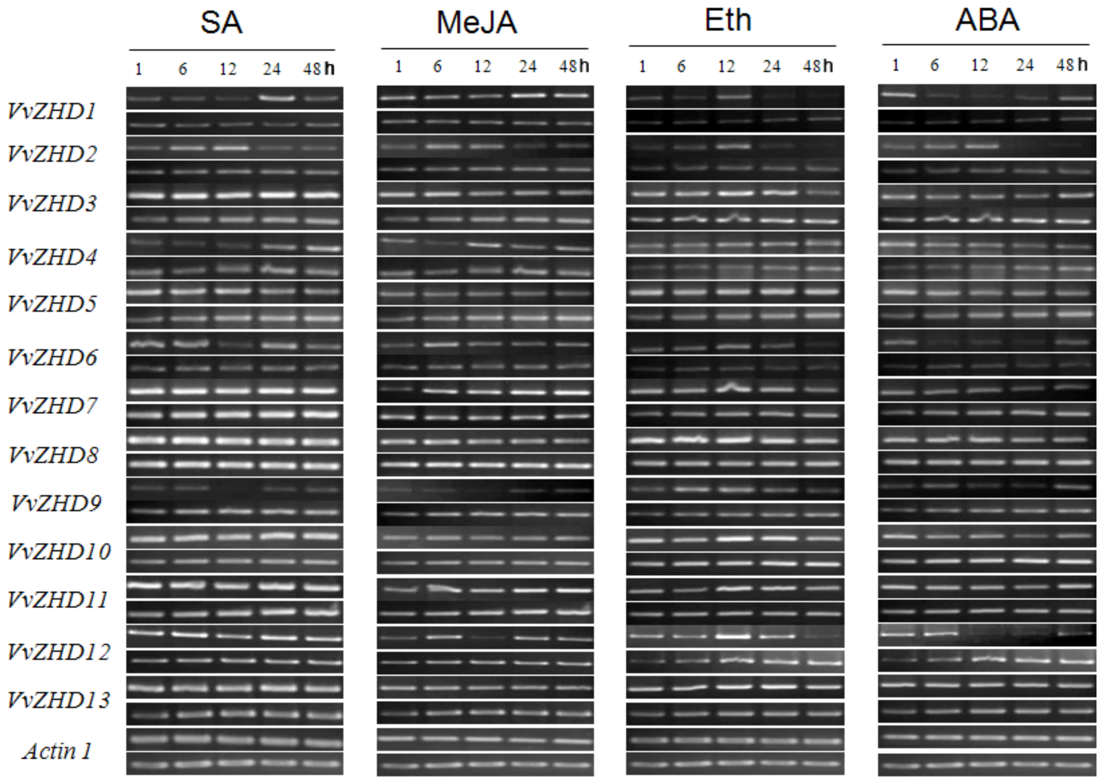

Table S1. The synteny regions and protein sequence alignment *E*-value between grape *ZHD* genes.

ID	Region 1 (Grape gene 1)			Region 2 (Grape gene 2)			Gene in the synteny region		
	Chr	Start	End	Chr	Start	End	Gene 1	Gene 2	*E*-value
1	chr18	10068356	12470528	chr4	17429918	20086815	*VvZHD11*	*VvZHD3*	1.00×10^{-54}
2	chr1	7420883	15243545	chr14	27319398	29948353	*VvZHD2*	*VvZHD8*	1.00×10^{-66}

Table S2. The synteny regions and protein sequence alignment *E*-value between grape and *Arabidopsis ZHD* genes.

ID	Region 1 (Arabidopsis gene 1)			Region 2 (Grape gene 2)			Gene in the synteny region		
	Chr	Start	End	Chr	Start	End	Gene 1	Gene 2	*E*-value
1	at5	26101939	26177917	chr18	4806872	5850896	*AtZHD1*	*VvZHD10*	2.00×10^{-44}
2	at5	15893761	15958455	chr1	12223982	11409315	*AtZHD10*	*VvZHD2*	1.00×10^{-22}
3	at1	26086948	26600219	chr1	13718634	7333255	*AtZHD11*	*VvZHD2*	3.00×10^{-42}
4	at5	17125578	17272164	chr18	10597337	11316066	*AtZHD13*	*VvZHD11*	2.00×10^{-19}
5	at1	5001011	5212349	chr1	6605920	4084591	*AtZHD14*	*VvZHD1*	2.00×10^{-24}
6	at4	12617256	12725637	chr18	6452788	5343830	*AtZHD2*	*VvZHD10*	2.00×10^{-55}
7	at2	621155	700917	chr12	22605027	22011079	*AtZHD3*	*VvZHD7*	3.00×10^{-56}
8	at1	28157530	28524337	chr18	10033200	12971832	*AtZHD5*	*VvZHD11*	4.00×10^{-48}
9	at1	28209437	28266124	chr4	18049833	18592042	*AtZHD5*	*VvZHD3*	7.00×10^{-44}
10	at2	7911054	8079360	chr18	11042060	10227177	*AtZHD6*	*VvZHD11*	2.00×10^{-49}
11	at2	7634274	8079360	chr4	20841170	17612788	*AtZHD6*	*VvZHD3*	3.00×10^{-48}
12	at3	18834967	19097353	chr4	18074949	20711919	*AtZHD7*	*VvZHD3*	3.00×10^{-37}
13	at3	10620627	11269229	chr14	28486389	30137019	*AtZHD9*	*VvZHD8*	4.00×10^{-52}

Table S3. The Percent Identity of 13 *VvZHD* genes ClustalX alignment.

ID	Gene name	Percent (%)												
1	*VvZHD3*	100.00	78.07	76.11	78.95	72.81	73.68	65.79	65.79	65.79	60.36	54.39	53.51	50.00
2	*VvZHD11*	78.07	100.00	74.34	77.19	75.44	73.68	64.91	61.40	64.91	60.36	49.12	50.00	48.21
3	*VvZHD4*	76.11	74.34	100.00	85.84	73.45	75.22	69.03	61.95	62.83	56.36	53.98	53.10	45.95
4	*VvZHD7*	78.95	77.19	85.84	100.00	76.32	74.56	64.04	64.91	64.04	58.56	51.75	51.75	47.32
5	*VvZHD10*	72.81	75.44	73.45	76.32	100.00	86.84	65.79	64.04	63.16	55.86	48.25	47.37	50.00
6	*VvZHD13*	73.68	73.68	75.22	74.56	86.84	100.00	69.30	64.91	64.91	54.05	50.00	50.00	47.32
7	*VvZHD12*	65.79	64.91	69.03	64.04	65.79	69.30	100.00	62.07	64.66	54.95	52.63	52.63	50.00
8	*VvZHD2*	65.79	61.40	61.95	64.91	64.04	64.91	62.07	100.00	88.79	59.46	46.49	45.61	52.68
9	*VvZHD8*	65.79	64.91	62.83	64.04	63.16	64.91	64.66	88.79	100.00	59.46	48.25	48.25	52.68
10	*VvZHD9*	60.36	60.36	56.36	58.56	55.86	54.05	54.95	59.46	59.46	100.00	51.35	53.15	53.57
11	*VvZHD5*	54.39	49.12	53.98	51.75	48.25	50.00	52.63	46.49	48.25	51.35	100.00	88.60	49.11
12	*VvZHD6*	53.51	50.00	53.10	51.75	47.37	50.00	52.63	45.61	48.25	53.15	88.60	100.00	47.32
13	*VvZHD1*	50.00	48.21	45.95	47.32	50.00	47.32	50.00	52.68	52.68	53.57	49.11	47.32	100.00

Table S4. Primer sequences used for semi-quantitative RT-PCR and quantitative real-time PCR of the 13 grape Zinc Finger-Homeodomain (*VvZHD*) genes. The specific primers were designed based on the *VvZHD* gene sequences using the Primer 5.0 software.

Gene ID	Gene locus ID	Primers	
		**Forward primer (5' to 3')**	**Reverse primer (5' to 3')**
VvZHD1	XM_002281662.1	ACCGCCGTCACACCATCATT	TGTTGTTGTTCAGCCAAGTCCT
VvZHD2	XM_002273766.1	TTCGCACCAAGTTCAGTCAAGAG	GCTGTCGCCATTGATATTGTTGA
VvZHD3	XM_002266541.2	ACCGTCACCTTCATCATCACCAA	GAATTGAGGCTGCTGATTGAGGAG
VvZHD4	XM_002267711.1	AGCAGCAGTGAGGTACAGAGAA	GAAGATGGCCGAAGGTGTAGGA
VvZHD5	XM_002264255.1	AGGAACGAAGAAGCCGAGAACA	GGTGGCGATGCTGATGATGAG
VvZHD6	XM_002264214.1	CGAAGGAAGAAGCCAAGAACAA	TGAGCAGAAGGTGGGAGAAGTG
VvZHD7	XM_003633364.1	TGGTGGTGGCGGTGTTGTT	TCTTGGCTAGGTTGTGCTTGTTG
VvZHD8	XM_002283497.2	CTGCTGCTGTTGTTGCTGAT	GGTGTTGTTGGTGCCTCTTC
VvZHD9	XM_002263430.1	AGGAAGGAGGAAGAGGACGAGAT	AGTGGTAGAAGCAGAAGACGAAGA
VvZHD10	XM_002285673.1	AGGAACACGAAGAACAGGAGGAA	GCGGTGGAAGTTGCGATGAC
VvZHD11	XM_002281335.2	AAGAACCACGCAGCCAACATC	CTCTGAGATCCTCGCTTGAAGACT
VvZHD12	XM_002276544.1	GAGGATGAAGCCGCCGAGAA	GGAACACCGAGGAACTTATGAGGA
VvZHD13	XM_003635009.1	TCAAGAAGAGCACGAGGAAGAGAT	TGGTGGTGATGGTGGAAGCC

© 2014 by the authors; licensee MDPI, Basel, Switzerland. This article is an open access article distributed under the terms and conditions of the Creative Commons Attribution license (http://creativecommons.org/licenses/by/3.0/).
